# Lisinopril-Induced Acute Necrotizing Pancreatitis

**DOI:** 10.7759/cureus.14642

**Published:** 2021-04-23

**Authors:** Salman B Syed, Jonathan Vincent M Reyes, Muhammad Baig

**Affiliations:** 1 Internal Medicine, University of Illinois College of Medicine at Peoria - OSF Saint Francis Medical Center, Peoria, USA; 2 Internal Medicine, Icahn School of Medicine at Mount Sinai, Elmhurst Hospital Center, Elmhurst, USA; 3 Gastroenterology, University of Illinois College of Medicine at Peoria - OSF Saint Francis Medical Center, Peoria, USA

**Keywords:** lisinopril, acute pancreatitis, necrotizing pancreatitis, pancreas, ace inhibitor induced pancreatitis, angiotensin converting enzyme inhibitor, drug induced pancreatitis, lisinopril induced pancreatitis

## Abstract

Lisinopril is an angiotensin-converting enzyme (ACE) inhibitor that is used as one of the first-line antihypertensive medications. Necrotizing pancreatitis induced by the use of ACE inhibitors is an extremely rare occurrence. Although an uncommon risk factor, our aim is to further highlight that patients with chronic use of lisinopril can develop such complications and should be considered among the list of differential diagnoses for pancreatitis.

A 53-year-old Caucasian male with a history of hypertension treated with lisinopril presented with a one-day history of nausea, vomiting, and severe epigastric pain. On physical examination, there was tenderness to palpation in the epigastric region and left lower quadrant without rebound tenderness or guarding. A complete blood count showed a slight increase in white blood cell count to 12,000 cells/mm^3^ and serum lipase level was elevated at 1028 U/L. A subsequent CT scan of the abdomen with contrast revealed findings supporting necrotizing pancreatitis. The patient was treated with conservative medical management with goal-directed intravenous fluid support, early enteral feeding, and pain control. His condition resolved, and he was found doing well on follow-up visits.

## Introduction

About less than 0.1-2.0% of total acute pancreatitis cases have been related to drug use [1]. Diagnosis of drug-related pancreatitis is difficult to establish. In addition, a causal relationship is difficult to determine as patients are not re-challenged with the drug due to ethical considerations and due to the risk of inducing a life-threatening situation [2]. However, recurrent acute pancreatitis secondary to the repeated use of lisinopril has been reported in the literature [3].

To the best of our knowledge, based on an extensive review of the literature using PubMed, Google Scholar, and Medline only one other case has been reported about necrotizing pancreatitis with the use of lisinopril [4]. The exact mechanisms for angiotensin-converting enzyme (ACE) inhibitor-induced pancreatitis are unknown; however, many theories have been proposed. It is known that ACE inhibitors increase serum bradykinin concentrations, the mechanism linked to ACE-induced angioedema. A local accumulation of bradykinin may lead to both angioedema and acute pancreatitis. Therefore, it is thought that they may also cause localized angioedema in the pancreas, resulting in pancreatic duct obstruction. Some studies suggest that they may cause an increase in pancreatic enzymes (amylase and lipase) production as well as the formation of antibodies directed against the pancreatic cells [2]. ACE inhibitors are antihypertensives that may also precipitate pancreatic ischemia [4].

As the rate of hospitalizations related to acute pancreatitis in the United States continues to rise, it is imperative for physicians to diagnose offending agents to reduce the overutilization of our healthcare system [5]. Gallstones and alcohol use are the two most common causes of acute pancreatitis. The other causes of pancreatitis are rare and sometimes mislabeled as idiopathic. An accurate diagnosis will prevent readmission, decrease mortality, and provide the medical community with answers to unsolved questions.

## Case presentation

A 53-year-old male presented to the emergency room with a one-day history of nausea, vomiting, and epigastric pain. The patient’s pain was severe in intensity and located in the mid-epigastric region with no reported abdominal trauma. His pain was associated with constant nausea and two episodes of non-bilious and non-bloody emesis episodes. He denied any febrile episodes, gastrointestinal overt bleeding, and chronic weight loss. He did not have any history of cigarette smoking, alcohol abuse disorder, or illicit drug usage. His past medical history was significant for essential hypertension being treated with oral lisinopril 20 mg daily. On physical examination, he was hemodynamically stable with body temperature, blood pressure, and heart rate of 37.3 °C, 140/90 mmHg, and 84 beats/min, respectively. Abdominal examination revealed tenderness to palpation in the epigastric region without rebound tenderness or guarding. At this point, our differential diagnosis was broad which included viral gastroenteritis, peptic ulcer disease, acute pancreatitis, acute cholecystitis, and choledocholithiasis.

**Figure 1:**
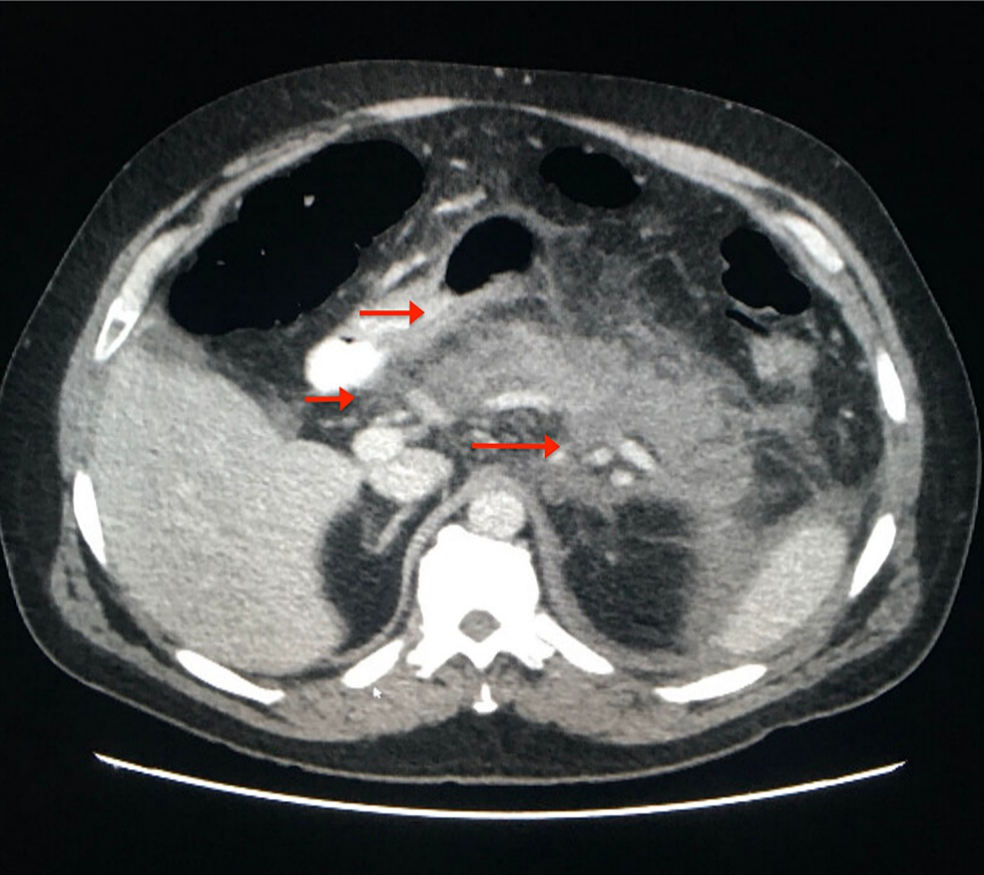
Diffuse hypoattenuation of the pancreatic parenchyma with peripancreatic fat stranding. Non-enhancing homogeneous fluid adjacent to the tail of the pancreas and within the left pararenal space (red arrows).

The following investigations were normal or negative: complete metabolic panel, urinalysis, lipid profile, electrocardiogram, and chest X-ray. A complete blood count showed a slight increase in white blood cell count to 12,000 cells/mm^3^ and serum lipase level was elevated at 1028 U/L. Calcium levels were 9.5 mg/dl (within the reference range), triglycerides were 54 mg/dl (reference for age <150 mg/dl), blood ethanol levels within normal limits, HIV test was nonreactive and IgG4 test was negative. Ultrasound of the abdomen showed no acute sonographic abnormality, no gallstones, and no ductal dilation. A CT scan of the abdomen showed pancreatic inflammation and pancreatic necrosis (Figure 1).

Diagnosis of acute necrotizing pancreatitis was established on the basis of physical examination, elevated serum lipase levels, and imaging findings, and conservative management was initiated with goal-directed intravenous fluid support (Lactated Ringer's at 250 ml/hr), early enteral feeding, and pain control. Diet was cautiously advanced over the course of hospitalization. As lisinopril was thought to be the possible cause of necrotizing pancreatitis, it was added to his list of allergic medications, and a different class of antihypertensive medication was commenced. 

Outcome and follow up

The patient showed gradual clinical improvement and was discharged home on day 7. He was noted to be asymptomatic on subsequent outpatient visits.

## Discussion

This report highlights that acute necrotizing pancreatitis can occur within therapeutic dose ranges of Lisinopril. Lisinopril-induced necrotizing pancreatitis is very rare; in addition to this case, there is only one other case reported based on extensive literature review. Literature reveals a variable onset of ACE inhibitor-induced pancreatitis. In this patient, the onset of pancreatitis was chronic, occurring after nine months of drug administration. Alternative causes such as alcohol use, gallstones, hypertriglyceridemia, blunt or penetrating abdominal trauma, autoimmune disorders, hypercalcemia, infection, anatomic, and genetic causes were excluded secondary to the patient’s history and laboratory findings. Therefore, a diagnosis of drug-induced pancreatitis was established.

The exact mechanism of this drug-induced pancreatitis has not yet been established; however, theories exist within the literature in the form of case reports, animal studies, and other observational data. Some of these theories include bradykinin accumulation, direct toxic effect, and upregulation of matrix metalloproteinase 9 (MMP-9) [6,7]. ACE inhibitors decrease the degradation of bradykinin which is linked to the known rare complication of angioedema. Bradykinin, a potent vasodilator, has been observed to cause localized angioedema around the pancreatic duct and increase vascular permeability allowing enzymes and toxic substances to invade the pancreas causing inflammation [1,2,8]. The theory that upregulation of MMP-9 leads to ACE inhibitor-induced pancreatitis comes from an animal study by Chen et al. The hypothesis is that MMP-9 also increases vascular permeability and increases the expression of transforming growth factor-beta which is involved in the regulation of inflammation, especially in the gut [9].

The severity of drug-induced pancreatitis varies and can range from mild to life-threatening. The exact incidence is unclear and potentially much higher due in part to possible poor recognition, especially for mild cases. Management of drug-induced pancreatitis includes discontinuation of the drug in question and supportive care.

## Conclusions

As the common causes for pancreatitis were excluded, we concluded that the cause of our patient’s acute pancreatitis was secondary to his lisinopril use. Ethically, this patient did not undergo a re-challenge and lisinopril was placed under the allergy list. Lisinopril, a commonly prescribed medication for hypertension, should not be overlooked when doing a medication reconciliation during hospital admission for acute pancreatitis. This case report will serve as evidence for this rare complication and add to the existing literature regarding ACE inhibitors and necrotizing pancreatitis. Physicians should always consider drug-induced pancreatitis as part of the differential diagnosis for acute pancreatitis.

## References

[1] Jones MR, Hall OM, Kaye AM, Kaye AD (2015). Drug-induced acute pancreatitis: a review. Ochsner J.

[2] Gorsane I, Ayed TB, Aoudia R, Kaaroud H, Hamida FB, Harzallah A, Abdallah TB (2019). Simultaneous acute pancreatitis and angioedema associated with angiotensin-converting enzyme inhibitor. Saudi J Kidney Dis Transpl.

[3] Kanbay M, Selcuk H, Yilmaz U, Boyacioglu S (2006). Recurrent acute pancreatitis probably secondary to lisinopril. South Med J.

[4] Bedrossian S, Vahid B (2007). A case of fatal necrotizing pancreatitis: complication of hydrochlorothiazide and lisinopril therapy. Dig Dis Sci.

[5] Gapp J, Hall AG, Walters RW, Jahann D, Kassim T, Reddymasu S (2019). Trends and outcomes of hospitalizations related to acute pancreatitis: epidemiology from 2001 to 2014 in the United States. Pancreas.

[6] Arakawa M, Murata Y, Rikimaru Y, Sasaki Y (2005). Drug-induced isolated visceral angioneurotic edema. Intern Med.

[7] Muchnick JS, Mehta JL (1999). Angiotensin-converting enzyme inhibitor-induced pancreatitis. Clin Cardiol.

[8] Misirlioglu M, Yildizdas D, Ekinci F, Horoz OO, Yontem A (2020). A rare cause of pediatric acute pancreatitis: perindopril intoxication. Turk J Emerg Med.

[9] Chen P, Yuan Y, Wang S, Zhan L, Xu J (2006). Captopril, an angiotensin-converting enzyme inhibitor, attenuates the severity of acute pancreatitis in rats by reducing expression of matrix metalloproteinase 9. Tohoku J Exp Med.

